# Targeting Epigenetic Regulators for Endometrial Cancer Therapy: Its Molecular Biology and Potential Clinical Applications

**DOI:** 10.3390/ijms22052305

**Published:** 2021-02-25

**Authors:** Futaba Inoue, Kenbun Sone, Yusuke Toyohara, Yu Takahashi, Asako Kukita, Aki Hara, Ayumi Taguchi, Michihiro Tanikawa, Tetsushi Tsuruga, Yutaka Osuga

**Affiliations:** Department of Obstetrics and Gynecology, Faculty of Medicine, The University of Tokyo, 7-3-1 Hongo Bunkyo-ku, Tokyo 113-8655, Japan; futabainoue0315@gmail.com (F.I.); yusuketoyo@gmail.com (Y.T.); cherrypon11@gmail.com (Y.T.); abibi1203a@gmail.com (A.K.); akiy925@gmail.com (A.H.); ayumikidu246@gmail.com (A.T.); tanikawa-tky@umin.ac.jp (M.T.); tsurugatetsushi@gmail.com (T.T.); yutakaos-tky@umin.ac.jp (Y.O.)

**Keywords:** epigenetics, DNA methylation, histone modification, histone acetylation, histone methylation, endometrial cancer

## Abstract

Endometrial cancer is one of the most frequently diagnosed gynecological malignancies worldwide. However, its prognosis in advanced stages is poor, and there are only few available treatment options when it recurs. Epigenetic changes in gene function, such as DNA methylation, histone modification, and non-coding RNA, have been studied for the last two decades. Epigenetic dysregulation is often reported in the development and progression of various cancers. Recently, epigenetic changes in endometrial cancer have also been discussed. In this review, we give the main points of the role of DNA methylation and histone modification in endometrial cancer, the diagnostic tools to determine these modifications, and inhibitors targeting epigenetic regulators that are currently in preclinical studies and clinical trials.

## 1. Introduction

### 1.1. Endometrial Cancer

Endometrial cancer (EC) is one of the most common gynecological malignancies [1]. Its incidence and mortality have been increasing all over the world. The American Cancer Society estimates about 65,620 new cases and about 12,590 deaths from cancer of the uterus in 2020. The mortality rate from this disease has been increasing for the past 20 years. The five-year relative survival rate of EC detected in the early stage is 95%; however, it is only 17% upon diagnosis in the advanced stages [2]. According to histological classification, type I tumors such as endometrioid carcinoma, comprise over 70% of EC cases and are related to high estrogen levels. In the early stages of type I tumors, hormone therapy can preserve fertility and patients often have a good prognosis. Type II tumors account for approximately 10% of EC cases that are not related to high estrogen, such as serous and clear cell carcinoma. Patients with type II tumors have poor prognosis even in early stage, having a recurrence rate of more than 50% [3]. Conventionally, the Bokhman’s dualistic model has been widely proposed to classify EC into type I and type II. In 2013, the Data from The Cancer Genome Atlas (TCGA) newly classified EC into four groups: *POLE* ultramutated, microsatellite instability (MSI) hypermutated, copy-number low, and copy-number high. Although the *POLE* ultramutated group (7%) has good prognosis, the copy-number high (serous-like cancers) group has poor prognosis [4,5]. The MSI hypermutated group and copy-number low (endometrioid cancers) group have intermediate prognosis. These molecular subtypes are critically important for prognosis and the choice of adjuvant treatment [4,6]. Considering the traditional classification, type I EC includes the low copy-number category often with *PTEN* mutations. Type II EC includes the high copy-number category frequently with p53 mutations. Although there are different types of EC, almost all patients diagnosed with EC undergo surgery (total hysterectomy, often along with a bilateral salpingo-oophorectomy), as a first treatment. Recently, laparoscopic hysterectomy and robot-assisted hysterectomy have also become widespread as minimally invasive surgery [7,8]. Younger woman with stage IA without myometrial invasion and grade 1 endometrioid EC may not remove their ovaries and may postpone the surgery during hormonal therapy. However, patients with high grade EC even in stage I and in an advanced stage undergo surgery in combination with pelvic and paraaortic lymphadenectomy [9]. Patients with high risk after surgery or in an advanced stage are commonly considered for chemotherapy with or without radiation therapy. However, since there are few optional treatments for recurrent patients, it is often refractory. Pembrolizumab can be used as an additional treatment in patients with high microsatellite instability (MSI high, MSI-H); however, there are no approved targeted therapies in EC to date [2,3]. Further understanding of epigenetic mechanisms is important to develop optional treatments in EC [10].

### 1.2. Epigenetics (DNA Methylation, Histone Modification, Non-Coding RNA)

To date, the study of genetics has been thought to be important in determining the phenotypes of diseases, especially after completion of the Human Genome Project in 2003 [11,12]. However, we often found that patients with the same type of cancer, staging, and genetic variants follow different mechanisms of chemotherapy resistance and prognoses in clinical settings. Hence, we cannot explain the phenotypes of diseases completely through single genetic variants [12,13]. Cancer is now considered not only a genetic disease but also an epigenetic disease. In fact, many researchers have attempted to shift to new directions in investigating the mechanisms behind cancer initiation and progression [12].

**Figure 1:**
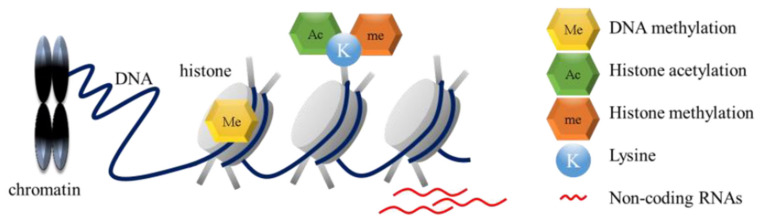
Epigenetic mechanisms. Epigenetic regulation involves DNA methylation, histone modification, and the effects of non-coding RNAs in regulating gene expression. In chromatin, DNA and histone proteins form nucleosomes, which are its functional repeating units. A histone octamer consists of two core histones (H2A, H2B, H3, and H4) and is wrapped around by 147 base pairs of DNA.

Epigenetics is the study of heritable changes in gene function that do not involve alterations in the DNA sequence [14,15]. Generally, epigenetic regulation involves DNA methylation, histone modification, and the effects of non-coding RNAs in regulating gene expression [11,16]. In chromatin, DNA and histone proteins form nucleosomes, which are its functional repeating units. A histone octamer consists of two core histones (H2A, H2B, H3, and H4) and is covered by 147 base pairs of DNA [11,17] (Figure 1).

**Figure 2:**
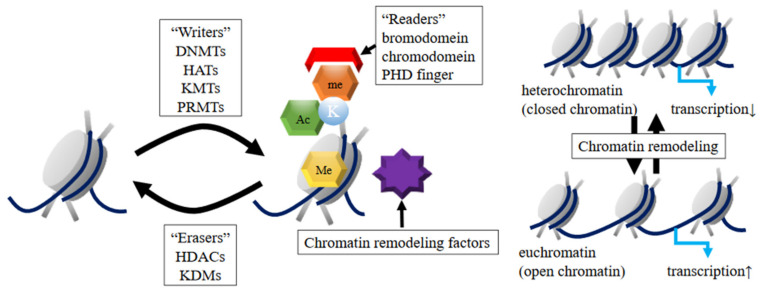
Epigenetic modification factors: “writers”, “readers”, and “erasers” and chromatin remodeling. The “writers” are enzymes that add chemical groups to DNA and histones. The “erasers” are also enzymes that remove chemical groups from the DNA and histones. The “readers” are proteins containing different motifs, which recognize distinct modifications and recruit additional chromatin modifiers and remodeling factors to affect the chromatin structure (chromatin remodeling). The chromatin structure can change between heterochromatin (closed chromatin) or euchromatin (open chromatin). In general, euchromatin has DNA more accessible to transcriptional factors and other chromatin regulators. DNMTs, DNA methyltransferases; HATs, histone acetyltransferases; KMTs, lysine methyltransferases; PRMTs, protein arginine methyltransferases; HDACs, histone deacetylases; KDMs, histone-demethylating enzymes; PHD finger, plant homeodomain finger.

Epigenetic modifications are coordinated by three factors: “writers”, “readers”, and “erasers”. The “writers” are enzymes that add chemical groups to DNA and histones. In contrast, the “erasers” are also enzymes; however, they remove chemical groups from the DNA and histones. The “readers” are proteins containing different motifs, which recognize distinct modifications and recruit additional chromatin modifiers and remodeling factors to affect the chromatin structure (chromatin remodeling). This way, the chromatin structure can change between heterochromatin (closed chromatin) or euchromatin (open chromatin). In general, euchromatin has DNA more accessible to transcriptional factors and other chromatin regulators. These modifications play essential roles in the regulation of transcription, DNA repair, and replication [11,13,18] (Figure 2).

In cancer cells, alterations in epigenetic events can be seen even in the early stages, leading to the development of tumors and progression of the disease. Each tumor cell in a tumor tissue can contain various epigenetic changes [11,19]. Aberrant epigenetics are known to contribute to cell cycle regulation, signaling pathways, invasion, metastases, and chemotherapy resistance [11,13,16,20].

Considering the importance of epigenetics in cancer cells, a further understanding of epigenetic regulation enables us to investigate cancer development and the therapeutic potential of new drug candidates. In this review, we describe the two main epigenetic mechanisms in EC: DNA methylation and histone modification. We also provided a list of inhibitors targeting epigenetic regulators, as well as current clinical trials investigating the effect of these inhibitors in EC.

## 2. Mechanisms of Carcinogenesis and Cancer Progression Involving DNA Methylation in EC

### 2.1. DNA Methylation

DNA methylation is one of the most extensively studied mechanisms of epigenetic modifications. DNA methylation patterns, DNA methylation, and demethylation are regulated by specific enzymes, following certain rules, in mammals. These methylation patterns then affect gene transcription [19].

The DNA methylation process is catalyzed by enzymes of the DNA methyltransferase (DNMT, referred to as the “writers”) family, which transfer methyl groups from S-adenosyl-L-methionine (SAM) to cytosine (C) residues to form 5-mC. The DNMT family consists of five members: DNMT1, DNMT2, DNMT3A, DNMT3B, and DNMT3L [21]. The majority of DNA methylation occurs in CpG islands, in which C is followed by a guanine (G). In the human genome, approximately 70% of CpG islands are present in promoters, and methylation of these sites leads to transcriptional repression. Methylation of CpG islands in promoters plays an essential role in cancer [19,22,23].

Aberrant DNA methylation is often observed in various diseases such as infectious diseases, metabolic disorders, neurological disorders, immunological disorders, precancerous lesions, and cancer [19,24,25]. In cancer, DNA methylation is thought to be one of its hallmarks. Hypermethylation of the promoter and the CpG islands of tumor suppressor genes are associated with transcriptional repression, although hypomethylation at repeat sequences is linked to genomic instability [26]. Aberrant DNA methylation can also occur during the early phase of cancer, even in the precancerous stage [13,27]. These mechanisms contribute to cancer development and drug resistance. Recently, DNA methylation in cancer has been thought to help the diagnosis of cancer and predict patient response to treatment [13,28].

### 2.2. DNA Methylation and EC

DNA methylation is also a widely studied epigenetic alteration in EC [29,30,31]. It has been reported that hypermethylation of tumor suppressor genes occur in atypical endometrial hyperplasia [32]. Aberrant DNA methylation in EC happens not only in the early stage but also in the precancerous stage [32,33]. Consequently, EC cell growth, proliferation, and apoptosis all involve DNA methylation, which regulates gene expression [30].

Both DNMT1 and DNMT3B are overexpressed in type I EC, such as endometrioid carcinoma, although they are downregulated in type II EC [34,35]. Thus, the hypermethylation of promoters, of genes such as *MLH1* and *PTEN*, is more common in type I EC [31,36], whereas global hypomethylation and genomic instability are induced in type II EC [31,34]. These mechanisms might be related to the histological and clinical differences between type I and type II tumors [31]. To date, more than 50 promoters of tumor suppressor genes have been identified as hypermethylated, including the most common genes, *MLH1*, *PTEN*, *MGMT*, *RASSF1A*, *PR*, and *CDH1* [37,38]. To date, gene promoter hypermethylation has been more frequently reported in type I EC than in type II EC. In 2013, an integrated genomic, transcriptomic and proteomic characterization of 373 endometrial carcinomas using array- and sequencing-based technologies was performed. According to the report, minimal DNA methylation changes were found in most of copy-number high tumors (serous-like cancers) [4].

The gene *MLH1* was reported as the key factor of abnormal DNA mismatch repair and MSI. Loss of its function is related to Lynch syndrome, also known as hereditary nonpolyposis colorectal cancer syndrome (HNPCC) [39]. The MSI hypermutated TCGA category is characterized by *MLH1* promoter hypermethylation [4]. MSI was seen in approximately 30% of EC cases [4,40], and *MLH1* promoter hypermethylation, which causes gene silencing, was seen in 23–35% of EC cases and is deeply associated with the MSI-high EC; 83–98% of the MSI-high EC cases are positive for *MLH1* promoter hypermethylation [41,42]. This mechanism can be observed starting from the precancerous lesion, and then progresses to complete methylation during cancer [22,24].

In addition to *MLH1*, promoter hypermethylation of other genes such as *PTEN*, *MGMT*, and *RASSF1A* has been reported in tumors with MSI. *PTEN* is a tumor suppressor gene. Its mutation is seen in 11% of the copy-number high tumors (serous-like cancers) TCGA category, although in 84% of other endometrioid tumors [4,25]. Germline mutations in *PTEN* are also responsible for Cowden syndrome, an inherited disorder characterized by hamartomas and a high risk of developing certain cancers, such as in the breast, thyroid, and uterus. *PTEN* promoter hypermethylation causes gene silencing and induces MSI [43].

*MGMT* is another DNA repair gene silenced by DNA methylation in 31% of EC [44]. Through the loss of MGMT function, DNA polymerases recognize O(6)-methylguanine as adenine. As a result, G to A mutations occur [45].

*RASSF1A* is a tumor suppressor gene, and *RASSF1A* silencing via promoter hypermethylation is frequently seen in EC and increased overall EC risk (*p* < 0.0001) [46]. However, it has been reported that *RASSF1A* can also be methylated in cervical adenocarcinoma (33.3%) [47]. Hence, DNA methylation may not be specific to certain cancers although it may represent histological subtypes [32].

In addition to the promoter methylation of the common genes mentioned above, a recent study reported that the status of promoter hypermethylation for *BCL2L11*, an apoptosis-associated gene, was correlated with malignant EC grade [48].

In contrast to type I EC, there are few reports regarding promotor hypermethylation in type II EC. Progesterone acts as a tumor suppressor in endometrial carcinogenesis and is used as hormonal therapy for hyperplasia and atypical hyperplasia [49]. The expression of the progesterone receptor (PR) is lower in type II EC than type I EC [36,50]. Loss of PR expression is observed in advanced EC patients and is associated with poor prognosis. Moreover, a higher percentage of PR promotor methylation was detected in type II EC cell lines than in type I EC cell lines (91% vs 6%) [51].

### 2.3. Diagnosis of Endometrial Cancer Using DNA Methylation

To date, the gold standard diagnostic tools for EC are surgical resection or the analysis of biopsy specimens [52,53]. However, false negatives caused by sampling errors can occasionally occur. In addition to biopsies, DNA methylation assays have been studied as novel diagnostic tools. Although diagnosis via the detection of aberrant methylation with serum is less invasive than traditional methods, the problem of this approach is depending on the presence of DNA from the tumor cells. It has been reported that abnormal methylation in primary tumors can be detected in serum in 42–76% of cases [54]. Abnormal methylation was also observed in samples close to primary tumor lesions, such as sputum for lung cancer, urine for bladder cancer, stool for colon cancer, and the lymph nodes for primary cancer [24]. Fiegl et al. [55] first investigated DNA methylation assays in EC using tampon tools, where they collected DNA samples from intravaginal tampons. DNA methylation of five genes, including *CDH13*, *HSPA2*, *MLH1*, *RASSF1A*, and *SOCS2*, were considered as diagnostic markers. If at least three of the five genes were hypermethylated, the sensitivity for detecting EC was 100% and its specificity was 91%. Further analyses using tampon methods have also been reported. Jamie et al. [56] found that the tampon method can be useful for detecting hypermethylated genes from endometrial tumors. They also identified that the methylation of three genes (*HTR1B*, *HOXA9*, and *RASSF1*) were significantly different between EC and benign endometrium. Sangtani et al. [57] reported that detecting the DNA methylation of *HOXA9*, *RASSF1*, and *HTR1B* for diagnosing EC have high specificities (100%) but low sensitivities (37–40%). However, they demonstrated that a combination of the analysis of copy number, DNA methylation, and gene mutation improved sensitivity (92%). In another study, combining methylation patterns in several genes (*BHLHE22*, *CDO1*, *TBX5*, and *HAND2*) with mutations in *PTEN* and *TP53* led to an endometrial cancer diagnosis with a sensitivity of 91.3% and a specificity of 42% [58].

The sensitivity and specificity of single gene DNA methylation assays are not yet enough to be used as a screening test [32]. However, the combination of hypermethylated genes and a combination of DNA methylation and other analyses might improve their sensitivity and specificity [32,57] and might provide a useful method for EC diagnosis in the future (Table 1).

Studies aimed at preventing endometrial cancer have also been reported. Nagashima et al. [60] compared DNA methylation signatures between obese patients who were asymptomatic but had a history of endometrial hyperplasia with normal endometrial cytology at the time of this study, and found similar DNA methylation signatures in several gene regions. This suggests that analysis of DNA methylation patterns may lead to the prevention of endometrial cancer.

## 3. Mechanisms of Carcinogenesis and Cancer Progression Involving Histone Modification in EC

### 3.1. Histone Modification

**Figure 3:**
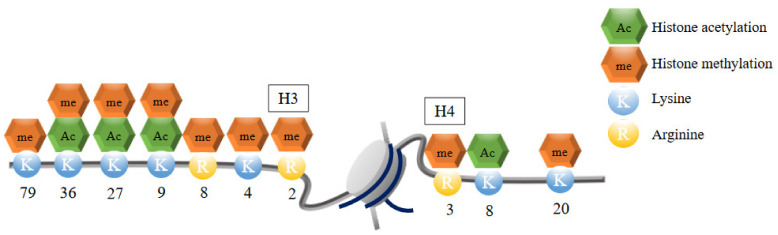
Important histone modification sites in H3 and H4. Histone modifications can occur at lysine and arginine residues on histone H3 or H4 tails. These modifications mainly include acetylation, methylation, phosphorylation, ubiquitylation, and sumoylation. Lysine residues can be mono-, di-, or tri-methylated (referred to as Kme1, Kme2, and Kme3), whereas arginine residues can be mono-, symmetrically or asymmetrically di-methylated (referred to as Rme1, Rme2s, and Rme2a). histone acetylation promotes gene transcription, although gene transcription regulated by histone methylation is more diverse and depends on the state of histone methylation.

Histone modifications can occur at lysine and arginine residues on histone tails [18,61] (Figure 3). Histone tails are targets of covalent posttranscriptional modifications (PTM). These modifications mainly include acetylation, methylation, phosphorylation, ubiquitylation, and sumoylation. Histone modifications at the promoters and enhancers are important for regulating gene expression. PTMs can add or remove chemical groups to modify histones and alter the chromatin structure [18,61,62]. creating either heterochromatin or euchromatin. Hence, appropriate PTMs are critical in human biology. Aberrant histone modifications are thought to be associated with tumorigenesis and occur at an early stage in cancer [63].

### 3.2. Histone Acetylation

Histone acetylation is also the well-studied mechanism of epigenetics, aside from DNA methylation [36,64]. Histone acetylation occurs through the addition of an acetyl group to the lysine residues in histone tails [65]. Although histone acetyltransferases (HATs, also referred to as “writers”) add acetyl groups, histone deacetylases (HDACs, referred to as “erasers”) remove acetyl groups. These changes are reversible and are usually involved in transcriptional regulation. PTM by HATs or HDACs plays an important role in various types of cancers. In EC, the deregulation of HDACs has been reported more than HATs [66].

#### 3.2.1. Histone Acetyltransferases (HATs)

Histone acetyltransferases are associated with active gene transcription at promoter and enhancer sites. In humans, there are about 30 HATs that are divided into three families: the general control nondepressible 5 (GCN5)-related N-acetyl transferase (GNAT) family (GCN5 and p300/CBP-associated factor [PCAF]), the MYST family (monocytic leukemic zinc factor [MOZ], MOZ-related factor [MOF], TIP60, and human acetylase binding to PRC1 [HBO1]), and the p300/CBP family (p300 and CBP) [67]. There are many other nuclear receptors and transcription factors aside from these HATs. HATs do not only catalyze histone acetylation but also protein acetylation outside histones [68].

#### 3.2.2. Histone Deacetylases (HDACs)

Histone deacetylases are generally associated with gene silencing and transcriptional repression. HDACs also catalyze both histone and non-histone proteins [18]. In humans, there are 18 HDACs that are classified into four groups [69]: Class I HDACs (HDAC1, 2, 3, and 8), Class II HDACs (HDAC4, 5, 6, 7, 9, and 10), Class III HDACs (SIRT1, 2, 3, 4, 5, 6, and 7), and Class IV HDACs (HDAC11). Class I, II, and IV HDACs have similar sequences and functional mechanisms. Aside from belonging to the HDAC family, Class III HDACs are also included in the Sir2 regulator family. Besides these differences, class I, II, and IV HDACs are Zn2^+^-dependent, whereas class III HDACs are NAD^+^-dependent [70,71].

#### 3.2.3. Histone Acetylation and EC

There are only a few reports about HATs in EC. Yi et al. [72] showed that MOF encoded by KAT8 can be a potential tumor suppressor that regulates estrogen receptor α (ERα) function in EC. Acetylation of H4K16 by MOF was suppressed using shMOF in EC cell lines.

In contrast to HATs, HDACs have been reported extensively in EC. HDACs regulate many cellular functions, including cell proliferation, apoptosis, and differentiation. Consequently, deregulation of HDACs is involved in tumorigenesis, therefore, HDAC inhibitors are effective in various types of cancers. In EC, HDACs are overexpressed compared to the normal endometrium [64,73,74]. The expression levels of HDAC1, 2, and 3 are higher in EC compared to that in normal endometrium, and these have been associated with poor prognosis [64,73]. Although many HDACs are overexpressed in EC, SIRT6 expression levels are lower in EC, inducing apoptosis by repressing survivin [75]. Similar to SIRT6, the expression levels of SIRT1, 2, 4, and 5 are downregulated in EC, whereas SIRT7 is overexpressed [76]. A recent study showed that SIRT1 expression is higher in endometrioid carcinoma (type I EC) than in clear cell carcinoma (type II EC). High SIRT1 expression in patients with EC is associated with better overall survival and progression-free survival than patients with low SIRT1 expression. This indicates that SIRT1 may be a tumor suppressor [77] (Table 2).

### 3.3. Histone Methylation

Histone methylation occurs through the transfer of methyl groups to the arginine or lysine residues in histone tails from S-adenosylmethionine (SAM) as a methyl donor. Similar to histone acetylation, histone methylation is regulated through the addition of methyl groups by histone methyltransferases (HMTs are referred to as “writers”) and the removal of methyl groups by histone demethylases (HDMs are referred to as “erasers”). Lysine residues can be mono-, di-, or tri-methylated (referred to as Kme1, Kme2, and Kme3), whereas arginine residues can be mono-, symmetrically or asymmetrically di-methylated (referred to as Rme1, Rme2s, and Rme2a). Compared to histone acetylation, which promotes gene transcription, gene transcription regulated by histone methylation is more diverse and depends on the state of histone methylation [11,18,78]. For instance, lysine methylation of H3K4, H3K36, and H3K79 are associated with active gene transcription, whereas other lysine methylations such as that in H3K9 and H3K27 are associated with silent gene transcription [78].

#### 3.3.1. Histone Methyltransferases (HMTs)

Unlike histone acetylation, which promotes gene transcription, histone methylation regulates both active gene transcription and gene repression. At promoter sites, H3K4me3 is associated with active genes, whereas H3K9me3 is associated with silent genes. At enhancer sites, H3K4me1 is associated with active genes. Appropriate histone methylation is critical for regulating gene expression [11,18].

HMTs can be divided into two groups: histone lysine methyltransferases (HKMTs) and protein arginine methyltransferases (PRMTs). HMTs also catalyze methyl transfer not only in histone proteins but also in non-histone proteins [18,79,80]. To date, there have been more studies on HKMTs than PRMTs.

Many studies have reported that histone methylation by HKMTs is linked to cancer development. Over 100 KMTs have already been identified in the human genome. Lysine methylation occurs mainly on histone H3 or H4. HKMTs can be classified into six groups according to the major histone methylation sites: H3K4 is methylated by SETD1A, SETD1B, MLL1, MLL2, MLL3, MLL4, SETD7, and PRDM9; H3K9 is methylated by SUV39H1, SUV39H2, SETDB1, and G9a, also known as EHMT2, and GLP, also known as EHMT1; H3K36 is methylated by SETD2, NSD1, NSD2, NSD3, and ASH1L; H3K79 is methylated by DOT1L; H4K20 is methylated by SET8, SUV420H1, and SUV420H2; and H3K27 is methylated by EZH1 and EZH2. In addition to these sites and enzymes, there are several other lysine methylation sites, which are not well studied, such as H3K23me3 and H3K63me3 [79]. HKMTs are generally selective, except for PRDM9, which does not only methylate H3K4, but also H3K9 and H3K36. From the perspective of the domain that determines enzyme activity, all HKMTs contain a SET (suppressor of variegation, enhancer of Zeste, trithorax) domain that characterizes the activity of the enzyme. However, unlike other HKMTs, DOT1L is the only HKMT that does not contain the SET domain. Although histone modification usually occurs in histone tails, DOT1L catalyzes the methylation of H3K79 in the core domain of histones [79].

In humans, nine PRMTs have already been identified. Several studies report that arginine methylation by PRMTs is involved in tumorigenesis through gene transcription, X chromosome inactivation, DNA repair, and mRNA splicing [80]. Based on the types of methylation, PRMTs can be divided into three classes: type I PRMTs (PRMT1, PRMT2, PRMT3, coactivator-associated arginine methyltransferase 1 [CARM1/PRMT4], PRMT6, and PRMT8) that generate asymmetric dimethylarginine (ADMA); type II PRMTs (PRMT5 and PRMT7), that form symmetric dimethylarginine (SDMA); and type III PRMTs, such as PRMT7, which generate monomethylarginine (MMA). PRMT9 has not yet been classified into any of the three classes [80] (Table 3 and Table 4).

#### 3.3.2. Histone Demethylases (HDMs)

Histone lysine demethylases (HDMs) can be divided into two groups: the amine-oxidase type lysine-specific demethylases (LSDs), including LSD1, also known as KDM1A, and LSD2, also known as KDM1B, and the highly conserved JumonjiC (JMJC) domain-containing histone demethylases, including JHDM1, JHDM2 (JMJD1), JHMD3 (JMJD2), JARID, PHF, and UT families [18]. JMJD6, also a member of the JMJC family, acts as a histone lysine arginine demethylase of H3R2 and H4R3 [81]. LSD1, which was first identified as an HDM by Shi Y et al. [82] in 2004, mono- or di-demethylates H3K4 or H3K9. Overexpression of LSD1 is related to tumorigenesis and poor prognosis in diseases such as breast cancer [83], prostate cancer [84], and ovarian cancer [85] (Table 5).

#### 3.3.3. Histone Methylation and EC

Aberrant histone modification is often associated with various cancers [79,80]. Qing et al. [86] investigated the association between histone methylations (H3K27me3, H3K4me3, and H3K4me2) and clinicopathological data using immunochemistry. Expression levels of H3K4me2 and H3K4me3 are higher in EC than in normal endometrium and are associated with the degree of malignancy in endometrial tissues. The expression level of H3K27me3 did not differ between EC and normal endometrium [86]. However, Krilla et al. [87] and Oki et al. [88] observed that EZH2, which is responsible for H3K27me3, was overexpressed in EC compared to that in normal endometrium and is associated with poor prognosis. Dysregulation of H3K27me3 by EZH2 was found in various types of cancers [86,87]. G9A, also known as EHMT2, is overexpressed in EC and is associated with deep myometrial invasion. In EC cell lines, EHMT knockdown increased the level of E-cadherin upon H3K9me2 hypomethylation and decreased the recruitment of the CDH1 promoter DNA methyltransferase [89]. NSD2, also known as MMSET or WHSC1, is overexpressed in EC compared with normal endometrium and its expression is associated with clinicopathologic grade of the disease. NSD2 overexpression is also significantly correlated with poor prognosis [90]. ASH2L, a component of the histone methyltransferase complex, is also overexpressed in EC. High expression of ASH2L is associated with poor prognosis, whereas ASH2L knockdown suppressed EC cell growth by regulating PAX2 transcription and altering the levels of H3K4me3 and H3K27me3 [91,92,93,94,95]. LSD1, also known as KDM1A, has higher expression levels in EC than in normal endometrium, and is associated with poor prognosis [92,93,94]. The mechanisms of LSD1 in EC involve the PIK3K/AKT pathway through demethylation of H3K9me2 at the cyclin D1 promoter [94]. There are a few reports about arginine methylation by PRMTs in EC, whereas dysregulation of PRMTs has been reported in many human cancers [80]. Jiang et al. [91] reported that the expression of PRMT6 was higher in EC than in normal endometrium and is associated with poor prognosis via the AKT/mTOR pathway.

## 4. Inhibitors Targeting Epigenetic Regulators

### 4.1. Inhibitors Targeting DNA Methylation

There have been several preclinical studies of DNA methylation inhibitors for patients with EC; however, are still no clinical studies for this class of drugs in humans [96]. This section introduces some preclinical studies of these inhibitors in vitro. In these studies, DNA methylation inhibitors were administered to EC cell lines to determine their ability to inhibit cell growth and analyze the downstream genes regulated by DNA methylation. For example, administration of the DNA methylation inhibitor, 5-AZA, to EC cell lines resulted in a decreased expression of β-catenin and cyclin D1, as well as suppression of cell proliferation [97]. The DNA methylation inhibitor RG 108 inhibited DNMT3B in an EC cell line, promoting demethylation of the mismatch repair gene *hMLH1* and inducing apoptosis [60]. Furthermore, addition of estrogen (E2: estradiol) to an EC cell line resulted in an increased expression of DNMT3B and enhanced cell proliferation, whereas administration of an estrogen antagonist (ICI282780) suppressed DNMT3B expression [98]. This suggests that estrogen increases the expression level of DNMT3B and can be involved in carcinogenesis and the progression of EC, and that antagonizing and inhibiting estrogen may be a therapeutic target for DNA methylation. Furthermore, several reports have indicated therapeutic advantages with combination therapy consisting of DNMT and HDAC inhibitors. It is well studied that HDAC inhibitors may not only regulate histone acetylation, but also modify DNA methylation. The combination therapy of an HDAC inhibitor and a DNMT inhibitor cause synergistic inhibition of cell growth in EC cell lines and mouse xenografts. Therefore, HDAC inhibitors and DNA methylation inhibitors are considered to be effective as a combination treatment [99,100,101]. Based on the above evidence, DNA methylation inhibitors may be effective against EC, so clinical trials involving these molecules are awaited.

### 4.2. Inhibitors Targeting Histone Modification

#### 4.2.1. Histone Deacetylase Inhibitors and the Treatment of EC

Based on their molecular mechanisms, HDAC inhibitors are divided into four groups: short-chain fatty acids, such as phenylbutyrate (PB), valproic acid (VPA), and carboxylic acids NaB; hydroxamic acids, including trichostatin A (TSA) and suberoylanilide hydroxamic acid (SAHA, vorinostat); benzamides, such as entinostat (MS-275) and mocetinostat (MGCD-0103); and cyclic peptides, such as romidepsin (FK228). In addition to these four groups, an increasing number of HDAC inhibitors have been developed [18,62,102]. According to previous studies, there are two major mechanisms of HDAC inhibitors: to control cellular functions, such as cell cycle arrest, cell growth inhibition, as well as apoptosis, and to promote the expression of tumor suppressor genes by regulating gene transcription [62,102]. To date, some HDAC inhibitors that are anticancer drugs, such as romidepsin and vorinostat, have been approved by the FDA for other malignancies [103]. Most clinical trials of HDAC inhibitors in phase I/II are for patients with hematologic malignancies and other cancers [18,104]. There is only one clinical trial for EC, which is in phase I (ClinicalTrials.gov identifier NCT03018249) that evaluates the effect of a combination HDAC inhibitors, Entinostat (MS-275) and medroxyprogesterone acetate therapy (Table 6). Several preclinical studies have also been reported for the treatment of EC. HDAC inhibitors have been reported to induce apoptosis in EC cell lines; however, different mechanisms of apoptosis induction have been studied. Romidepsin (FK228), an HDAC inhibitor, induces apoptosis in EC cells by activating the p53-p21 pathway [105]. Entinostat (MS-275) increases p21 and p27 expression and promotes apoptosis [106]. Trichostatin A (TSA) and apicidin both suppress cell growth and induce p21 expression and apoptosis in EC cell lines [73]. Several studies have investigated the combination of HDAC inhibitors and other anticancer agents, such as carboplatin, docetaxel, gemcitabine, cisplatin, etoposide, doxorubicin, and paclitaxel in gynecological cancers [107,108]. Combination therapy with TSA and paclitaxel enhances inhibition of cell growth in EC cell lines [103]. In another report, a combination of the HDAC inhibitor OBP-801/YM753 and a PI3K inhibitor induced apoptosis in EC cells by increasing Bim expression with increased ROS generation [109]. HDAC inhibitors have also been reported to restore the expression of progesterone receptors in EC cells. Moreover, it was proven that HDAC inhibitors suppressed oncogene MYC in EC cells [110]. Vorinostat is also effective in EC cell lines. It is considered that the insulin-like growth factor (IGF) system is related to the carcinogenesis of EC, and that vorinostat was found to induce apoptosis by suppressing its IGF signals [111]. In addition, the SIRT inhibitor, MHY2256, reduced the expression level of SIRT1, 2, and 3, and significantly inhibited EC tumor growth in vivo through p53 acetylation [112].

#### 4.2.2. Inhibitors of Histone Methyltransferases (HMTs) and Histone Demethylases (HDMs) and the Treatment of EC

There are several inhibitors targeting HMTs and HDMs, such as DOT1L inhibitors, EZH2 inhibitors, and LSD1 inhibitors. Ongoing clinical trials for these molecules are approved mainly for hematological malignancies. However, a phase I clinical trial of LSD inhibitor (SP-2577) for EC (ClinicalTrials.gov identifier NCT04611139) has been started in October 2020. This is a study of the combination therapy of the LSD inhibitor (SP-2577) and the anti PD-1 antibody pembrolizumab in patients with EC (Table 6). Since there are fewer studies about histone methylation than histone acetylation in EC, there have been few preclinical studies, most of which involve EZH2 inhibitors and LSD1 inhibitors. Ihira et al. [113] reported that the addition of GSK343, an EZH2 selective inhibitor, to EC cell lines suppressed cell growth through increased expression of miR-361 and decreased expression of Twist. Real-time PCR showed increased expression of EZH2 in EC clinical specimens, and tissue microarrays showed a negative correlation between EZH2 expression and disease prognosis. Both EZH2 knockdown and administration of the EZH2 inhibitor, GSK126 suppressed the proliferation of EC cell lines and significantly induced apoptosis [88]. In another report, EZH2 knockdown also increased the expression SERP1, DKK3, and E-cadherin and decreased β-catenin expression [114].

The existence of H3K9 methyltransferase and EHMT2 (G9a) inhibitors, have also been reported. Here, the addition of an EMHT2 inhibitor to EC cell lines resulted in the suppression of cell proliferation in vitro as well as myometrial invasion by repressing E-cadherin [89].

The LSD1 inhibitor, HCI2509, suppressed cell growth in type II EC cell lines and mouse xenograft models, inducing apoptosis. Elevation of H3K27me3 was also observed using an LSD1 inhibitor [93].

The analysis considering histone modification was not carried out in each report. In fact, mechanisms that regulate downstream genes via acetylation and methylation are conceivable. It is therefore necessary to understand the detailed antitumor mechanism of these inhibitors when considering their potential clinical applications. Analysis of histone modifications, such as ChIP-seq and ATAC-seq, should be performed in the future.

## 5. Conclusions

Epigenetic mechanisms are critical in normal biological function as well as tumorigenesis. In the past 20 years, epigenetics research has made remarkable progress, and new analyses such as ATAC-seq and ChIP-seq have also been discovered. However, there are few studies regarding the epigenome in EC. Therefore, it is necessary to deepen our understanding of epigenetic mechanisms so it could be useful in the development of therapeutic drugs and the expansion of indications of current anticancer drugs to include EC. However, there are some problems that need to be overcome. First, the epigenetic mechanisms in EC are still unknown. Even though DNA methylation has been extensively studied, there are still few reports regarding histone modification. As we have previously mentioned here, further analyses, such as ChIP-seq and ATAC-seq in EC, are needed to identify new downstream genes and develop novel biomarkers. Second, it is also important to elucidate the epigenetic mechanisms in the four molecular prognostic categories proposed by the TCGA. Although DNA methylation data from the four TCGA categories has already been revealed, to date, there are no specific reports regarding histone modifications. These aspects could help the development of novel therapeutic drugs. Although there are only two inhibitors targeting the epigenome for EC currently in clinical trials, it would be possible to expand the therapeutic potential of not only a single agent but also combination therapies with existing drugs. Although further preclinical studies and evaluation of adverse events for epigenetic inhibitors will be needed, there is hope in discovering new drugs targeting the epigenome in EC.

## Figures and Tables

**Table 1:** Classification of DNA methyltransferase and endometrial cancer.

Enzyme	Endometrial Cancer
DNMT1	DNMT1 is overexpressed in type I EC, although it is downregulated in type II EC [34,35].
DNMT2	
DNMT3A	
DNMT3B	DNMT3B is overexpressed in type I EC, although it is downregulated in type II EC [34,35]. DNMT3B inhibition promotes demethylation of hMLH1, inducing apoptosis [59].
DNMT3L	

**Table 2:** Classification of major histone modification factors for histone acetylation and endometrial cancer.

	Enzyme	Synonym	Endometrial Cancer
Histone acetyltransferases (HATs): writers			
GNAT family	GCN5	KAT2A	
	PCAF	KAT2B	
MYST family	MOZ	MYST3, KAT6A	
	MOF	MYST1, KAT8	MOF regulates ERα function as a tumor suppressor in EC [72].
	TIP60	HTATIP, KAT5	
	HBO1	MYST2, KAT7	
p300/CBP family	p300	EP300, KAT3B	
	CBP	CREBBP, KAT3A	
Histone deacetylases (HDACs): erasers			
Class I HDACs	HDAC1		The expression levels of HDAC1, 2, and 3 are higher in EC compared to that in normal endometrium, and these have been associated with poor prognosis [64,73].
	HDAC2		
	HDAC3		
	HDAC8		
Class II HDACs	HDAC4		
	HDAC5		
	HDAC6		
	HDAC7		
	HDAC9		
	HDAC10		
Class III HDACs	SIRT1		The expression levels of SIRT1, 2, 4, and 5 are downregulated in EC [76]. SIRT1 expression is higher in endometrioid carcinoma (type I EC) than in clear cell carcinoma (type II EC). High SIRT1 expression in patients with EC is associated with better prognosis [77].
	SIRT2		
	SIRT3		
	SIRT4		
	SIRT5		
	SIRT6		SIRT6 is downregulated in EC, inducing apoptosis by repressing survivin [75].
	SIRT7		SIRT7 is overexpressed in EC [76].
Class IV HDACs	HDAC11		

**Table 3:** Classification of major histone methyltransferases (lysine) and endometrial cancer.

Substrate	Enzyme	Synonym	Endometrial Cancer
Lysine methyltransferases (KMTs)			
H3K4	SETD1A	KMT2F	
	SETD1B	KMT2G	
	MLL1	KMT2A	
	MLL2	KMT2B	
	MLL3	KMT2C	
	MLL4	KMT2D	
	SETD7	KMT7, SET7, SET7/9	
	PRDM9	PFM6	
H3K9	SUV39H1	KMT1A	
	SUV39H2	KMT1B	
	SETDB1	KMT1E	
	G9A	EHMT2, KMT1C	EHMT2 is overexpressed in EC and is correlated with deep myometrial invasion and cell proliferation. EHMT2 inhibition induces apoptosis [80,81,82,83,84,85,86,87,88,89].
	GLP	EHMT1	
H3K36	SETD2	KMT3A, SET2	
	NSD1	KMT3B, ARA267	
	NSD2	KMT3G, WHSC1, MMSET	NSD2 is overexpressed in EC and is associated with poor prognosis. NSD1 expression is correlated with clinicopathologic grade of the disease [90].
	NSD3	KMT3F, WHSC1L1	
	ASH1L	KMT2H, ASH1	
H3K79	DOT1L	KMT4	
H4K20	SET8	KMT5A, SETD8	
	SUV420H1	KMT5B	
	SUV420H2	KMT5C	
H3K27	EZH1	KMT6B, KIA0388	
	EZH2	KMT6, KMT6A	EZH2 is overexpressed in EC compared to normal endometrium and is associated with poor prognosis [87,88].

**Table 4:** Classification of histone methyltransferases (arginine) and endometrial cancer.

Type	Products	Enzyme	Endometrial Cancer
Protein arginine methyltransferases (PRMTs)			
type I PRMTs	asymmetric dimethylarginine (ADMA)	PRMT1	
		PRMT2	
		PRMT3	
		PRMT4/CARM1	
		PRMT6	Overexpression of PRMT6 is associated with poor prognosis via AKT/mTOR pathway [91].
		PRMT8	
type II PRMTs	symmetric dimethylarginine (SDMA)	PRMT5	
		PRMT7	
type III PRMT	monomethylarginine (MMA)	PRMT7	
PRMT9 has not yet been classified.			

**Table 5:** Classification of major histone demethylases (HDMs) and endometrial cancer.

	Enzyme	Synonym	Endometrial Cancer
LSD (KDM1)	LSD1	KDM1A	LSD1 is overexpressed in EC and is associated with cell proliferation [92,93,94]. LSD1 expression is correlated with poor prognosis, involving the PIK3K/AKT pathway through demethylation of H3K9me2 at the cyclin D1 promoter [94].
	LSD2	KDM1B	
JHDM1 (KDM2)	JHDM1A	KDM2A	
	JHDM1B	KDM2B	
JHDM2 (JMJD1, KDM3)	JHDM2A	KDM3A	
	JHDM2C	KDM3C	
JHMD3 (JMJD2, KDM4)	JHDM3A	KDM4A	
	JMJD2B	KDM4B	
JARID (KDM5)	JARID1A	KDM5A	
	JARID1B	KDM5B	
PHF (KDM7)	JHDM1F	KDM7B	
UT (KDM6)	UTX	KDM6A	
	JMJD3	KDM6B	

**Table 6:** Ongoing clinical trials with inhibitors targeting epigenetic regulators for endometrial cancer.

NCT03018249	
Condition	FIGO Grade 1 endometrial endometrioid adenocarcinoma FIGO Grade 2 endometrial endometrioid adenocarcinoma FIGO Grade 3 endometrial endometrioid adenocarcinoma
Design	HDAC inhibitor, Entinostat (MS-275) + medroxyprogesterone acetate
Phase	I
Sample size	50
Recruitment status	Active, not recruiting
First Posted Date	12 January 2017
**NCT04611139**	
Condition	small cell ovarian cancer of the hypercalcemic type ovarian clear cell tumor ovarian endometrioid adenocarcinoma endometrial cancer
Design	LSD inhibitor, Seclidemstat (SP-2577) + PD-1 antibody, Pembrolizumab
Phase	I
Sample size	30
Recruitment status	Not yet recruiting
First Posted Date	2 November 2020
